# Microbe Profile: Candida glabrata – a master of deception

**DOI:** 10.1099/mic.0.001518

**Published:** 2024-11-26

**Authors:** Maria Granada, Emily Cook, Gavin Sherlock, Frank Rosenzweig

**Affiliations:** 1 School of Biological Sciences, Georgia Institute of Technology, Atlanta, GA 30332, USA; 2 Department of Genetics, Stanford University School of Medicine, Stanford, CA 94305-5120, USA

**Keywords:** *Candida glabrata*, candidiasis, opportunistic pathogen, yeast

## Abstract

*Candida glabrata* is a fungal microbe associated with multiple vertebrate microbiomes and their terrestrial environments. In humans, the species has emerged as an opportunistic pathogen that now ranks as the second-leading cause of candidiasis in Europe and North America (Beardsley *et al*. *Med Mycol* 2024, 62). People at highest risk of infection include the elderly, immunocompromised individuals and/or long-term residents of hospital and assisted-living facilities. *C. glabrata* is intrinsically drug-resistant, metabolically versatile and able to avoid detection by the immune system. Analyses of its 12.3 Mb genome indicate a stable pangenome Marcet-Houben *et al*. (*BMC Biol* 2022, 20) and phylogenetic affinity with *Saccharomyces cerevisiae*. Recent phylogenetic analyses suggest reclassifying *C. glabrata* as *Nakaseomyces glabratus* Lakashima and Sugita (*Med Mycol J* 2022, 63: 119-132).

## Graphical abstract

*Candida glabrata*, an opportunistic fungal pathogen. (a) Unable to form hyphae, relies on tissue disruption to invade and disseminate host. (b) Calcofluor-white cells, 60X objective. (c) Colony morphology on YPD.

## Taxonomy

*Candida glabrata* was first isolated from human stool by Anderson (1917), who designated this isolate as *Cryptococcus glabratus* [1]. When pseudohyphae formation was found to be an unreliable taxonomic character, the organism was reclassified as *Torulopsis glabrat*a [2]. It is currently classified as follows: domain: Eukaryota, kingdom: *Fungi*, subkingdom: *Dikarya*, phlyum: *Ascomycota*, subphylum: *Saccharomycotina*, class: *Saccharomycetes*, order: *Saccharomycetales*, family: *Saccharomycetaceae*, genus: *Nakaseomyces*, clade: *Nakaseomyces/Candida* and species: *glabratus*/*glabrata*. Reclassification to *Nakaseomyces glabratus* has been proposed, based on expanded molecular and phylogenetic analyses [3].

## Properties

*C. glabrata* is a haploid yeast averaging 3 µM in length that grows optimally at 37 °C and up to 42 °C. A facultative anaerobe, the species is auxotrophic for thiamine, pyridoxine and nicotinic acid [4]. As mating has never been observed, the organism has long been considered asexual [5]. However, recent genomic analyses provide evidence for mating-mediated recombination [6]. *C. glabrata* resides as a commensal on the epithelium of healthy individuals but can become pathogenic in those with a weakened immune system. As *C. glabrata* does not form pseudohyphae, it requires tissue barrier disruption to enter the bloodstream, where it can disseminate and evade the host immune system by invading macrophage.

## Genome and evolution

The first complete genome sequence of *C. glabrata* was published in 2004; a revised assembly was released in 2020 [7]. The 12.3 Mb genome harbours 5272 predicted ORFs, of which 571 ORFs (10.83%) have been experimentally verified. Lengths of *C. glabrata*’s 13 nuclear chromosomes range between 512 655 and 1 528 264 bp. The nuclear genome is dynamic as evidenced by extensive chromosomal length polymorphism due to translocations and tandem gene repeats. The *C. glabrata* mitochondrion encloses a 20 kb circular genome consisting of 11 ORFs. The most frequently studied laboratory strains are CBS138 (ATCC 2001) and BG2. CBS138 was isolated from human faeces [1] and was the first to be fully sequenced. BG2 was derived from the parental strain ‘B’, which was a vaginitis, fluconazole-resistant isolate [8].

*C. glabrata* is closely related to the non-pathogenic yeast, *Saccharomyces cerevisiae*. Both species belong to the whole-genome duplication (WGD) group, and gene order along their respective chromosomes is largely conserved [9]. A noteworthy difference between the two species is the expansion of adhesion genes in *C. glabrata* [10]; these are thought to be important virulence factors, as they enable the organism to adhere to different host substrates and facilitate biofilm development [11].

*C. glabrata* exhibits remarkable genomic plasticity that may play a role in antifungal resistance and pathogenicity. For example, sub-telomeric adhesin genes vary both in copy number and in DNA sequence among different *C. glabrata* strains [6,12]. Independent isolates may exhibit different karyotypes, inviting speculation that large-scale rearrangements may facilitate adaptation to novel environments. Analysis of sequential isolates among patients undergoing antifungal therapy has shown that chromosomal rearrangements occur alongside the evolution of drug resistance. Although a causal relationship between these events remains uncertain, genome plasticity has the potential to contribute to evolutionary adaptation [13].

## Phylogeny

*Candida* is a polyphyletic genus within the subphylum *Saccharomycotina,* which includes both CTG and non-CTG clades as well as pre- and post-WGD groups. *C. glabrata* and its sister taxa, including *Saccharomyces cerevisiae*, belong to the non-CTG clade that translates the CUG codon into leucine, similar to most eukaryotes. However, many other *Candida spp*. belong to the CTG (or CUG) clade, in which species translate CUG to serine [9]. While some have speculated that pathogenicity correlates with this coding shift, species with varying degrees of medical relevance exist within both clades [14]. In any case, differences within the *Saccharomycotina* in their genome sizes and coding rules likely played roles in protein diversification.

Multiple closely related pathogenic and nonpathogenic species exist within the *Nakaseomyces* clade, the newly proposed taxonomic home for *C. glabrata*. When examined as a group, these species could provide a valuable resource for experimental and comparative genomic studies aimed at discovering the molecular bases of virulence [5,6].

## Key features and discoveries

Little is known about the environmental reservoirs of *C. glabrata*, although the species has been recovered from settings as diverse as vertebrate hosts, including humans and birds [15], surfaces of flowers and leaves and water and soil [5]. In humans, *C. glabrata* can be a normal component of the epithelial microbiome of the skin, oral cavity, gastrointestinal tract and urogenital tract. Given this range of habitats, it is thought that *C. glabrata* can be found anywhere near human habitation.

*C. glabrata* is of particular clinical relevance because of its low susceptibility to azole drugs [12], which are clinically employed as a cost-effective, first-choice preventative and as a second-choice treatment for invasive candidiasis. As with other antibiotics, this practice has led to increased cross-resistance in *C. glabrata* to most types of azoles [16]. Additionally, *C. glabrata* exhibits the capacity to evolve secondary resistance to multiple antifungal drug classes (polyenes, echinocandins and flucytosine), especially after exposure to more than one drug [11,12, 17]. One means by which azole resistance is achieved is via mutations in pleiotropic drug resistance 1 (*PDR1*), a transcription factor that controls the regulation of drug efflux pumps [9]. As *PDR1* now appears to play a role in many cellular responses, it may be better characterized as a sensor of overall cell stress [18]. Regulation of *PDR1* was recently shown to be controlled by the chromatin remodeler SWI/SNF (SWItch/Sucrose Non-Fermentable ATP-dependent chromatin remodeling complex subfamily) and the histone chaperone Rtt106, as the deletion of these genes sensitizes cells to antifungal drugs. Because Rtt106 and several components of the SWI/SNF complex are fungal-specific, this discovery opens up the possibility of new therapeutic targets [19].

Drug resistance in *C. glabrata* may also be associated with the emergence of petite or small-colony variants (SCVs). This phenotype has been observed in azole and echinocandin resistance studies using *in vitro*, *in vivo* and patient isolates [20,22]. With low prevalence and of uncertain fitness value, much remains to be learnt about this phenotype’s clinical significance, warranting its surveillance.

*C. glabrata* can form biofilms, which appear to increase drug resistance and contribute to treatment failure. *C. glabrata* biofilms are compact structures composed of cells surrounded by an extracellular matrix consisting of proteins and carbohydrates. Biofilm-associated drug resistance is complex, as this matrix increases cell density, protects cells against drugs, upregulates genes encoding efflux pumps and favours the emergence of ‘persisters’, quasi-dormant cells that contribute to chronic infection [11].

To survive within its host, *C. glabrata* combines resistance and evasion strategies. For example, *C. glabrata* exhibits intrinsically high resistance to chemical stressors employed by the immune system such as increased osmolarity and reactive oxygen species and decreased pH [11]. Additionally, the yeast’s metabolic flexibility enables it to survive in nutrient-limited host environments [2]. One key genetic component is yapsins, a family of glycosylphosphatidylinositol (GPI)-linked aspartyl proteases that have been expanded in *C. glabrata* (relative to *S. cerevisiae*). These 11 genes are upregulated in macrophage infection assays [23] and are essential for cell wall integrity, virulence, glucose homeostasis and epithelial adherence [2,24].

*C. glabrata* employs immune evasion tactics that impair macrophage function and reduce inflammatory responses, enabling the yeast to survive and proliferate within immune cells. Macrophages initially locate yeast cells by identifying pathogen-associated molecular patterns. In *C. glabrata,* these include the cell wall polysaccharides chitin, *α*-mannan and *β*-glucan. Nutrients are severely limited inside macrophages, creating a hostile environment for the yeast once it has been engulfed [25]. However, once inside the macrophage’s phagosome, *C. glabrata* has the capacity to impair phagosome maturation [2], disrupting acidification and lysosomal pathways that would otherwise destroy engulfed cells. Macrophages harbouring *C. glabrata* also produce less pro-inflammatory cytokines, which are important in recruiting other immune cells [26]. Once phagosome maturation has been subverted, *C. glabrata* is free to replicate within a nonacidic phagosome [25], which ultimately leads to macrophage lysis due to high fungal loads. The orchestration of this process is not yet understood mechanistically. A *C. glabrata* mutant screen uncovered *mnn10* and *mnn11* deletions that failed to prevent vacuole acidification in an *ex vivo* assay [27], suggesting that mannosyltransferases impact protein glycosylation and secretion, which aid in neutralization. Yapsins also contribute to immune evasion [2,28]. Deletion of *YPS1*-*11* results in higher immune activation, correlated with differential regulation of cell wall metabolism, and increased exposure of cell wall chitin and adhesin Epa1. Recent work mapping nucleosome placement in *C. glabrata* during macrophage engulfment [28] revealed widespread chromatin changes; these include SWI/SNF-mediated remodelling of immunogenic cell wall components, which repressed full immune recognition and response.

In summary, *C. glabrata* is a highly adaptable, intrinsically drug-resistant organism of increasing medical relevance. Ultimately, a better understanding of how individuals acquire, host and transmit *C. glabrata* is needed to reduce risks to human health posed by this formidable opportunistic pathogen.

## Open questions

- To what extent do environmental reservoirs of *C. glabrata* serve as sources for human colonization?
- Does *C. glabrata* undergo meiosis and mating? And if so, under what conditions?
- In human hosts, does long-term residence in one environment (skin, oral cavity, gastrointestinal and urinary tracts) influence the propensity for *C. glabrata* to disseminate and/or develop specific patterns of antifungal drug resistance?
- What diagnostic tools would provide clinicians with higher sensitivity, specificity and faster turn-around times to manage *C. glabrata* infections?
- What types of surveillance can be implemented at the national and international levels to control the spread of antifungal drug resistance in *C. glabrat*a?

## Abbreviations

GPI: glycosylphosphatidylinositol
PAMPs: pathogen-associated molecular patterns
PDR1: pleiotropic drug resistance 1
SCVs: small-colony variants
SWI/SWNF: sucrose non-fermentable ATP-dependent chromatin remodeling complex subfamily
WGD: whole-genome duplication
